# Alpha Lipoic Acid Improves Endothelial Function and Oxidative Stress in Mice Exposed to Chronic Intermittent Hypoxia

**DOI:** 10.1155/2019/4093018

**Published:** 2019-04-09

**Authors:** Mohammad Badran, Bisher Abuyassin, Saeid Golbidi, Najib Ayas, Ismail Laher

**Affiliations:** ^1^Department of Anesthesiology, Pharmacology and Therapeutics, The University of British Columbia, Vancouver, Canada; ^2^Divisions of Critical Care and Respiratory Medicine, Department of Medicine, The University of British Columbia, Vancouver, BC, Canada; ^3^Sleep Disorders Program, UBC Hospital, Vancouver, BC, Canada; ^4^Division of Critical Care Medicine, Providence Health Care, Vancouver, BC, Canada

## Abstract

**Objective:**

Obstructive sleep apnea (OSA) is characterized by recurrent airway collapse that causes chronic intermittent hypoxia (CIH). OSA is associated with systemic inflammation and oxidative stress resulting in endothelial dysfunction and cardiovascular disease (CVD). Alpha lipoic acid (ALA) is a potent antioxidant with anti-inflammatory properties. We hypothesized that dietary ALA can improve endothelial function of mice exposed to CIH.

**Methods:**

Mice were exposed to either CIH or intermittent air (IA) and treated with dietary ALA (0.2% *w*/*w*) or a regular chow diet for 8 weeks. Endothelial function, endothelial nitric oxide (eNOS) uncoupling, systemic oxidative stress, systemic inflammation, aortic expression of inflammatory cytokines, and antioxidant enzymes were measured after 8 weeks.

**Results:**

Mice exposed to CIH exhibited endothelial dysfunction accompanied by systemic oxidative stress and inflammation as well as increased aortic expression of inflammatory cytokines. Furthermore, CIH led to eNOS uncoupling. Treatment with dietary ALA reversed endothelial dysfunction in mice exposed to CIH, lowered systemic oxidative stress and inflammation, prevented the increases of inflammatory cytokine gene expression, increased the expression of antioxidant enzymes, and preserved eNOS in a coupled state.

**Conclusion:**

ALA attenuates endothelial dysfunction by preventing oxidative stress and inflammation and restoring nitric oxide bioavailability in mice exposed to CIH. Our data suggests the potential beneficial use of ALA as adjunctive therapy in OSA.

## 1. Introduction

Obstructive sleep apnea (OSA) is characterized by repetitive collapse of the pharyngeal airway during sleep, leading to intermittent hypoxia followed by reoxygenation. These changes can activate several pathological pathways such as oxidative stress and inflammation that can lead to endothelial dysfunction and cardiovascular disease (CVD) [1]. Indeed, OSA is considered an independent risk factor for CVD, with cardiovascular events occurring three times more frequently in patients with OSA compared to controls [2]. Animals exposed to intermittent hypoxia (model of OSA) also experience cardiovascular and metabolic sequelae including oxidative stress, hypertension, glucose intolerance, and endothelial dysfunction [3].

Continuous positive airway pressure (CPAP) is the standard treatment in patients with moderate to severe OSA and improves sleepiness and reduces blood pressure [4]. However, 46 to 83% of OSA patients are nonadherent to CPAP [5]. Results from the recent SAVE study indicated that CPAP prescription did not prevent cardiovascular events in patients with moderate to severe OSA and established CVD when compared to those who received usual care alone [6]. This may in part be due to the relatively low adherence (less than 4 hours per night) to CPAP use. This strongly suggests that other treatments are needed to improve cardiovascular health in patients with OSA.

Alpha lipoic acid (ALA) is a naturally occurring dithiol compound enzymatically synthesized from octanoic acid in the mitochondria. ALA and its reduced form dihydrolipoic acid (DHLA) act as potent antioxidants though various pathways including reduction of oxidized endogenous antioxidants (vitamin C and glutathione) and modulation signaling pathways for nuclear factor kappa B (NF-*κ*B) and insulin [7]. ALA is a commonly used and readily available dietary supplement. ALA improves endothelial function in patients with type 2 diabetes [8] and Alzheimer's disease [9]. ALA also reduces oxidative stress and inflammation in numerous animal models of disease including atherosclerosis [10, 11]. We hypothesized that dietary ALA ameliorates endothelial dysfunction in mice subjected to chronic intermittent hypoxia (CIH) by attenuating oxidative stress and inflammation and restoring nitric oxide bioavailability.

## 2. Materials and Methods

### 2.1. Animals and CIH Protocol

Experimental protocols were approved by the Animal Care Centre at The University of British Columbia, Canada (certificate # A16-0291). Adult male C57BL/6 (10 weeks old) mice were purchased from Charles River (Wilmington, MA). Forty mice were divided into four groups of mice subjected to the following: (1) intermittent air (IARD) and fed with regular diet (Research Diets, New Brunswick, NJ), (2) IA and fed with diet containing 2% *w*/*w* Bio-Enhanced® Na R-Lipoic Acid (ALA, GeroNova Research, Richmond, CA) (IALA), dose was determined based on previous studies [12], (3) intermittent hypoxia (IHRD), and (4) IH and fed with diet containing ALA (IHLA). Details of the CIH protocol were described previously [13]. Briefly, mice were housed in customized cages with ports spaced evenly to allow for uniform airflow from all sides. A gas control delivery system regulated the flow of N_2_ and compressed air inside the cages. A combination of flow regulators, oxygen sensors, and programmable solenoid valves were used to control the fraction of oxygen inspired (*Fi*O_2_), which could be adjusted over a wide range of CIH profiles. During the 12-hour light cycle (mice are nocturnal animals), *Fi*O_2_ was reduced from 21% to 5-6% for 30 seconds using N_2_ gas followed by 30 seconds of compressed air, so returning *Fi*O_2_ back to 21%. This was repeated for a total of 60 cycles per hour for 8 weeks. The oxyhemoglobin saturation (SpO_2_) nadir at the end of hypoxic cycles reached 55-60%. A similar protocol was used for the control groups, where only compressed air was delivered (no N_2_). We allowed the mice to acclimate to the hypoxic stimulus by first setting the nadir *Fi*O_2_ at 18% and then gradually reducing every two days by 2% until it reached the desired experimental levels of 5-6% to allow the mice to acclimate to the hypoxic stimulus.

### 2.2. Biochemical Measures

Mice were euthanized according to the University Animal Care Centre (ACC) guidelines using the inhalant anesthetic isoflurane (5%) at 1-2 L O_2_/minute followed by carbon dioxide until the animals stopped breathing and were left in the chamber for at least 5 minutes after turning off the carbon dioxide. Fasting blood glucose was measured as previously described [14], and plasma insulin (ALPCO, Boston, MA), tumor necrosis factor alpha (TNF-*α*) (R&D Systems, Minneapolis, MI), and urinary 8-hydroxy-2′-deoxyguanosine (8-OHdG) (Cedarlane Laboratories, Burlington, ON) were all measured using enzyme-linked immunoassay (ELISA) according to manufacturer instructions.

### 2.3. Wire Myography

Segments of aortic rings (2 mm long) were mounted on a wire myograph for measuring isometric tension (DMT 620M, Danish Myo Technology, Aarhus, Denmark) as described previously [15]. Each myograph chamber contained physiologic salt solution (PSS) kept at 37°C and pH 7.4 with continuous administration of 95% O_2_ and 5% CO_2_ gases. High KCl solution was prepared by equimolar substitution of NaCl in PSS. Aortic rings were stretched to their optimal tension (5.5 mN), then left to equilibrate for 20 mins before being challenged with 80 mM KCl, and then returned to PSS again.

For endothelium-dependent vasodilation, aortic rings were preconstricted with a submaximal concentration of phenylephrine (Ph) (1 *μ*M) followed by cumulative additions of half-log concentrations (10^−10^-10^−5^ M) of acetylcholine (ACh). For determining the role of basal nitric oxide production, two consecutive Ph concentration response curves were made, first in the absence and then after incubation with the endothelial nitric oxide synthase (eNOS) inhibitor N*_ω_*-nitro-l-arginine methyl ester hydrochloride (L-NAME, 10^−4^ M). L-NAME inhibits eNOS to reduce intrinsic (basal) nitric oxide production, so causing a greater increase in Ph-induced vasoconstriction in proportion to the extent of basal nitric oxide produced. Basal nitric oxide production is estimated by the difference between the two PE curves and measured by the area under the curve (AUC) as we described elsewhere [15].

### 2.4. Staining for Endothelial Nitric Oxide Synthase (eNOS) Uncoupling

To investigate the role of eNOS as a source of superoxide anion, one of the two aortic rings from the same animal was incubated in L-NAME (500 *μ*M) for 30 mins at 37°C. Both rings were then embedded and cryosectioned (10 *μ*m). Sections were then incubated with the fluorescent superoxide-sensitive dye dihydroethidium (DHE) (1 *μ*M) (Molecular Probes) for 30 mins at 37°C in a humidity chamber. The reaction was stopped by placing the slides at 2-8°C for 20 mins. Slides were then coverslipped, and fluorescence was detected (absorbance: 518 nm, emission: 605 nm) using an Olympus BX61 microscope with a Retiga EXi camera (QImaging, Surrey, Canada), and images were analyzed by the corrected total cell fluorescence (CTCF) method using ImageJ software (NIH, Bethesda, MD).

### 2.5. Western Blotting

Fresh aortic tissues were homogenized in RIPA lysis buffer using a BeadBug homogenizer (Benchmark Scientific, Edison, NJ). Tissue homogenates were then transferred to sterilized filters with 0.8 *μ*m pores (Sartorius Stedim Biotech, Germany) and centrifuged for 10 minutes at 14000 × *g* to remove cell debris. Pierce bicinchoninic acid (BCA) assay was used to determine protein concentrations using manufacturer instructions (Thermo Fisher Scientific, Waltham, MA). For Western blotting, 30 *μ*g of samples protein was loaded on polyacrylamide gels at 200 volts for 1 hour. The gels were then removed and transferred to nitrocellulose membranes overnight at 4°C using Mini-PROTEAN Tetra cell (Bio-Rad Laboratories, Hercules, CA). Membranes were then incubated for 1 hour with 5% nonfat milk (New England Biolabs, Ipswich, MA) for blocking and then incubated with primary antibodies in tris-buffered saline with 0.05% Tween-20 (TBST) overnight at 4°C. The antibodies used were anti-DDAH2 (dimethylarginine dimethylaminohydrolase 2) rabbit monoclonal IgG at a dilution of 1 : 500 (Abcam, cat# ab184166), anti-4-HNE-modified protein (4-hydroxynonenal) rabbit polyclonal IgG (Abcam, cat# ab46545, RRID:AB_722490) at a dilution of 1 : 500, and anti-ALDH2 (aldehyde dehydrogenase 2) mouse IgG (Santa Cruz Biotechnology, cat# sc-100496, RRID:AB_2242451) at a dilution of 1 : 100. Following overnight incubation, membranes were washed for 15 minutes three times with TBS and the secondary goat anti-rabbit horseradish peroxidase-tagged antibody was added at a dilution of 1 : 2000 for DDAH2 and 4-HNE-modified proteins (Abcam cat# ab7090, RRID:AB_955417). The secondary mouse IgG kappa-binding protein was used for ALDH2 at a dilution of 1 : 1000 (Santa Cruz Biotechnology cat# sc‐516105, RRID:AB_2687626). After 1 hour of incubation at room temperature, detection was performed using an enhanced chemiluminescence kit Clarity Max (Bio-Rad, Hercules, CA).

### 2.6. Real-Time PCR for Gene Expression

RNA was extracted using QIAzol Lysis Reagent (QIAGEN, Hilden, Germany) and then purified using Isolated RNeasy Mini Kit (Qiagen, Hilden, Germany), according to manufacturer instructions. RNA quality and quantity were determined using Agilent 2100 Bioanalyzer (Agilent Technologies, Santa Clara, CA). Total RNA with ribosomal integrity number (RIN) > 7 were used for real-time PCR quantification by custom RT^2^ Profiler™ PCR Array (QIAGEN, Hilden, Germany).

Real-time PCR quantification was performed with an Applied Biosystems 7500 Real-Time PCR System (Applied Biosystems, Foster City, CA) using a custom-made RT^2^ Profiler PCR Array consisting of 14 genes, including glyceraldehyde 3-phosphate dehydrogenase (GAPDH) as a housekeeping gene. cDNAs were synthesized from 0.5 *μ*g of total RNA using a commercial RT^2^ First Strand Kit (QIAGEN, Hilden, Germany) according to manufacturer instructions. The synthesized cDNAs were then mixed with RT^2^ qPCR ROX master mix containing SYBR Green (QIAGEN, Hilden, Germany). The mixture was then added to the custom RT^2^ Profiler PCR Array, and qPCR was performed according to manufacturer instructions. Data was analyzed using the integrated web-based automated software for RT^2^ Profiler PCR Array Data analysis (RT^2^ Profiler PCR Array Data analysis version 3.5, GeneGlobe Data Analysis, SABiosciences). Gene expression fold changes were calculated using the ^ΔΔ^*C*_*T*_ method, and the housekeeping gene control was used for normalization of the results.

### 2.7. Statistical Analysis

Values are expressed as means ± SD (*n* = 4 − 10). Vascular function data were recorded and analyzed by PowerLab 4/25 and LabChart 7 Reader (ADInstruments, Australia). Relaxations are expressed as percentage changes in tension from the pre-contraction to Ph; contractions are expressed as percentage of the reference response to 80 mM KCL. Gene expression data were assayed in triplicates to ensure the reliability of single values, and statistical test was performed on ^ΔΔ^*C*_*T*_ values between groups, and data was expressed as fold regulation representing fold change in a biologically meaningful way. Two-way ANOVA with multiple comparisons followed by Bonferroni post hoc test was used to assess differences in the 4 groups; unpaired Student's *t*-tests were used for real-time PCR and within-group analysis of tissues before and after L-NAME in the DHE staining experiments using Prism version 6.0 (GraphPad Software, California, USA). A *P* value < 0.05 was considered significant.

## 3. Results

### 3.1. Basic Animal Characteristics at the End of the Experiment

As shown in Table 1, there were no significant changes in body weights, epididymal fat weight, and fasting blood glucose due to CIH or dietary ALA. However, plasma insulin levels were lower in the IHLA group when compared to the IHRD group (*P* < 0.05), likely due to the ability of ALA to improve insulin sensitivity [16].

### 3.2. ALA Improved Endothelial Dysfunction in Mice Exposed to CIH

**Figure 1:**
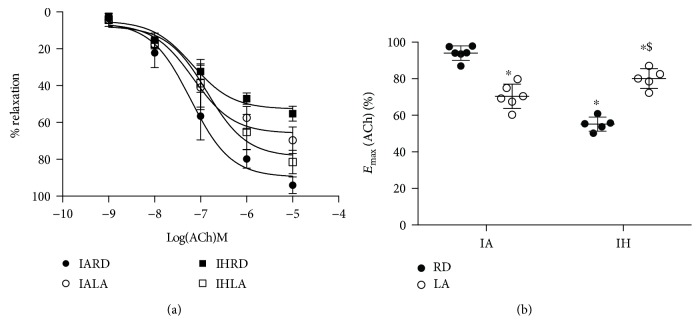
Endothelium-dependent relaxation in mice exposed to CIH and treated with ALA. Cumulative concentration response curves to acetylcholine (ACh) in aortic segments preconstricted with phenylephrine (Ph) (a) and maximum relaxation response to ACh (b). Values are shown as mean ± SD from 5-6 mice. Statistical analysis was performed by two-way repeated measures ANOVA followed by Bonferroni post hoc test. ^∗^*P* < 0.05 versus IARD, ^#^*P* < 0.05 versus IALA, and ^$^*P* < 0.05 versus IHRD.

Endothelium-dependent relaxation was reduced in mice subjected to CIH compared to control (*E*_max_: 55.2 ± 3.8% vs. 94.1 ± 4.3% of induced tone, *P* < 0.0001). Mice subjected to CIH and fed dietary ALA showed improved endothelial function when compared to CIH alone (*E*_max_: 80.1 ± 6.2%, *P* < 0.0001) (Figures 1(a) and 1(b)). Dietary ALA decreased relaxation in control mice subjected to intermittent air alone, probably due to ALA acting as an antioxidant or prooxidant depending on the oxidant levels and physiological status [17]. There were no significant changes in the EC_50_ for acetylcholine between all groups.

### 3.3. Treatment with ALA Restored Basal Nitric Oxide Production in Mice Exposed to CIH

**Figure 2:**
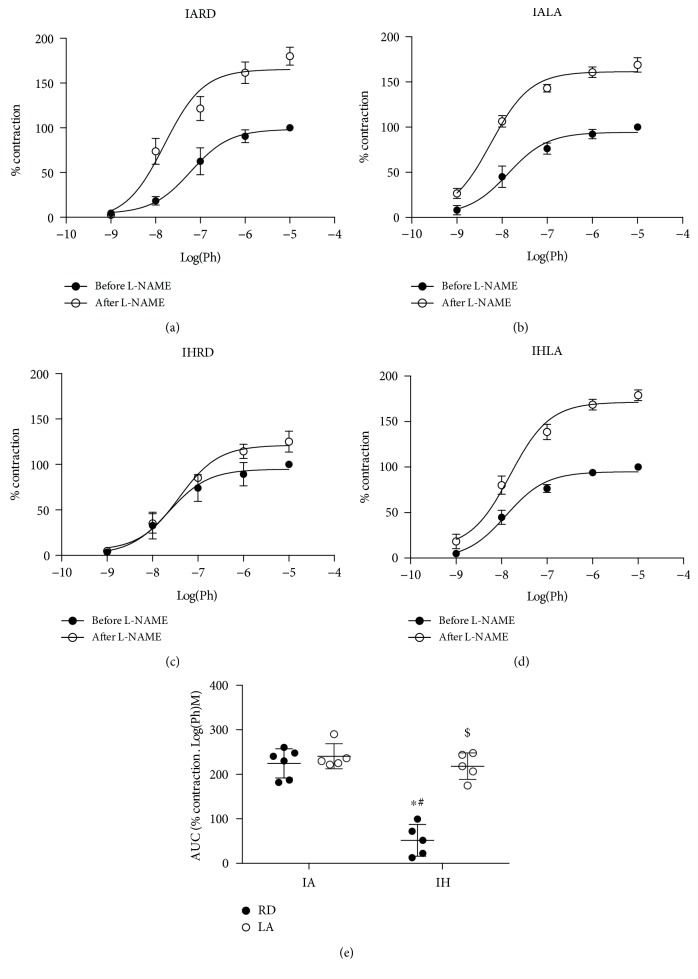
Basal NO production in mice exposed to CIH and treated with ALA. Cumulative concentration response curves to Ph before and after L-NAME (a–d). AUC calculated for the contraction response to Ph after adding L-NAME (e). Values are shown as mean ± SD from 5-6 mice. Statistical analysis was done by two-way repeated measures ANOVA followed by Bonferroni posttest. ^∗^*P* < 0.05 versus IARD, ^#^*P* < 0.05 versus IALA, and ^$^*P* < 0.05 versus IHRD. ACh: acetylcholine; AUC: area under the curve; L-NAME: N*_ω_*-nitro-L-arginine methyl ester; Ph: phenylephrine.

Basal production of nitric oxide maintains a vasodilatory tone in blood vessels at rest. Loss of that tone can lead to increase resting vasoconstriction and endothelial dysfunction. In the control and dietary ALA groups, the maximal contraction to phenylephrine was increased after incubation with L-NAME (% increase in *E*_max_: 179.8 ± 14.1 and 168.9 ± 9.1, respectively) (Figures 2(a) and 2(b)). The modest increases in the maximum response in mice subjected to CIH after incubation with L-NAME were restored by dietary ALA (% increase in *E*_max_: 123.1 ± 15.7 vs. 179.3 ± 8.1, *P* < 0.01) (Figures 2(c) and 2(d)). Basal nitric oxide production was attenuated in mice subjected to CIH but was restored by ALA treatment (Figure 2(e)).

### 3.4. Systemic Oxidative Stress and Inflammation Lowered in Mice Exposed to CIH and Treated with ALA

**Figure 3:**
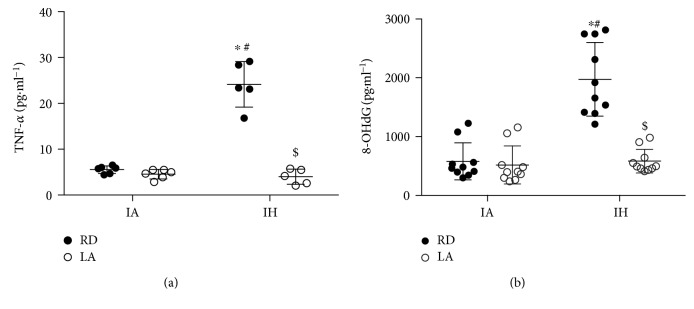
Plasma inflammatory and urinary oxidative stress markers. Plasma levels of TNF-*α* (a) and urinary levels of 8-OHdG (b). Values are displayed as mean ± SD from 5–10 mice. Statistical analysis was by two-way repeated measures ANOVA followed by Bonferroni post-hoc test. ^∗^*P* < 0.05 versus IARD, ^#^*P* < 0.05 versus IALA, and ^$^*P* < 0.05 versus IHRD. 8-OHdG: 8-hydroxy-2′-deoxyguanosine; TNF-*α*: tumor necrosis factor alpha.

Levels of urinary 8-OHdG, an oxidative stress marker for DNA damage, were higher in mice subjected to CIH when compared to control (1974.7 ± 627.1 vs. 578.7 ± 315.1 pg/mL, *P* < 0.0001) (Figure 3(a)). Dietary ALA decreased levels significantly in mice subjected to CIH (582.7 ± 201.6 pg/mL, *P* < 0.0001). Moreover, plasma levels of the inflammatory marker TNF-*α* were higher in mice subjected to CIH (24.1 ± 4.9 vs. 5.4 ± 0.1 pg/mL, *P* < 0.0001) and lowered after ALA treatment (4.0 ± 1.6 pg/mL, *P* < 0.0001) (Figure 3(b)).

### 3.5. ALA Preserves eNOS Coupling in Mice Subjected to CIH

**Figure 4:**
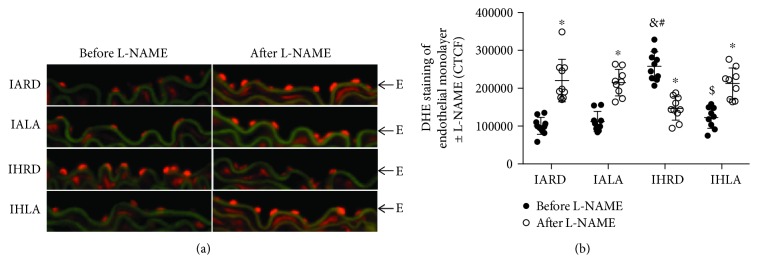
Uncoupled endothelial NOS in the endothelium. Representative images of dihydroethidium (DHE) staining of the endothelial monolayer before and after incubation with the endothelial NOS inhibitor L-NAME (20x magnification) (a). Quantification of fluorescence levels using corrected total cell florescence (CTCF) (b). Values are displayed as mean ± SD from 10 mice. Statistical analysis was done by using Student's *t*-test within groups before and after L-NAME. Two-way repeated measures ANOVA followed by Bonferroni post-hoc test was used for comparison between groups after L-NAME. ^∗^*P* < 0.05 before vs. after L-NAME, ^&^*P* < 0.05 versus IARD, ^#^*P* < 0.05 versus IALA, and ^$^*P* < 0.05 versus IHRD. CTCF: corrected total cell fluorescence; DHE: dihydroethidium; L-NAME: N*_ω_*-nitro-L-arginine methyl ester; NOS: nitric oxide synthase.

Oxidative stress transforms eNOS from a coupled (nitric oxide producing) to an uncoupled (superoxide producing) state. Under normal physiological conditions, basal production of superoxide is scavenged by nitric oxide. As seen in the endothelial layer of the control group (Figure 4(a)), increased florescence after incubation with the eNOS blocker (L-NAME) indicates prevention of superoxide scavenging by inhibiting nitric oxide production (CTCF: 100573 ± 22494 before L-NAME vs. 220384 ± 56462, *P* < 0.0001). However, decreased fluorescence after incubation with L-NAME in the mice subjected to CIH indicates eNOS uncoupling marked by reduced superoxide production (CTCF: 258053 ± 38225 vs. 146766 ± 30931, *P* < 0.0001). Treatment with ALA maintained eNOS in a coupled state since fluorescence was higher after incubation with L-NAME (CTCF: 122597 ± 28369 vs. 212614 ± 40729, *P* < 0.0001) (Figure 4(b)).

### 3.6. ALA Decreased Levels of ADMA in Mice Exposed to CIH

**Figure 5:**
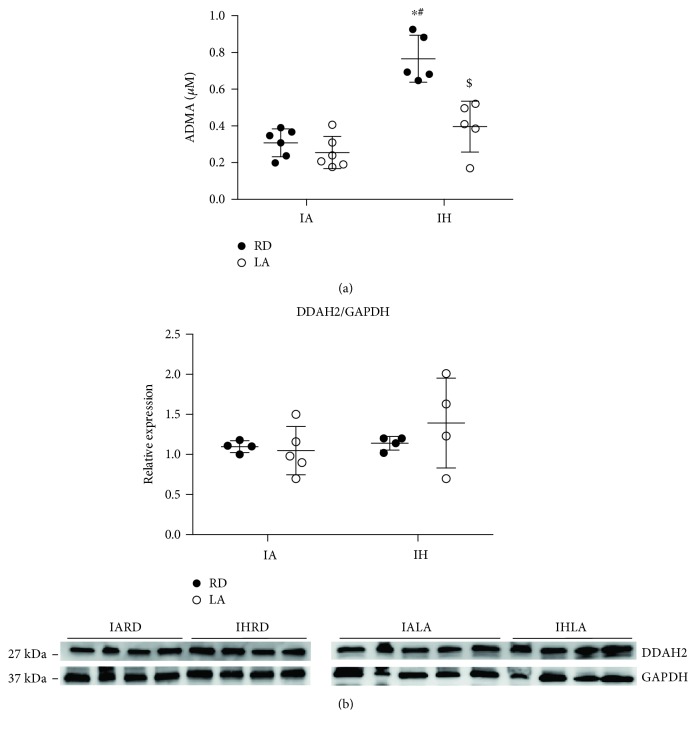
Levels of ADMA, an endogenous inhibitor of eNOS levels, and expression of DDAH2, its metabolizing enzyme. Plasma levels of ADMA (a) and aortic expression of DDAH2 (b). Values are displayed as mean ± SD from 5-6 mice for ADMA and 4-5 mice for DDAH2 expression. Statistical analysis was done by two-way repeated measures ANOVA followed by Bonferroni post-hoc test. ^∗^*P* < 0.05 versus IARD, ^#^*P* < 0.05 versus IALA, and ^$^*P* < 0.05 versus IHRD. ADMA: asymmetric dimethylarginine; DDAH2: dimethylarginine dimethylaminohydrolase 2; GAPDH: glyceraldehyde 3-phosphate dehydrogenase.

Plasma levels of ADMA, an endogenous competitive inhibitor of L-arginine [18], significantly increased in mice exposed to CIH when compared to control (0.76 ± 0.12 *μ*m vs. 0.31 ± 0.07 *μ*m, *P* < 0.0001). Treatment with ALA decreased ADMA levels (0.39 ± 0.13 *μ*m, *P* < 0.001) (Figure 5(a)). Changes in plasma ADMA levels occurred with no changes in the aortic expression of DDAH, the enzyme responsible for metabolizing ADMA (Figure 5(b)).

### 3.7. ALA Increased Levels of ALDH2 and Decreased Levels of 4-HNE

**Figure 6:**
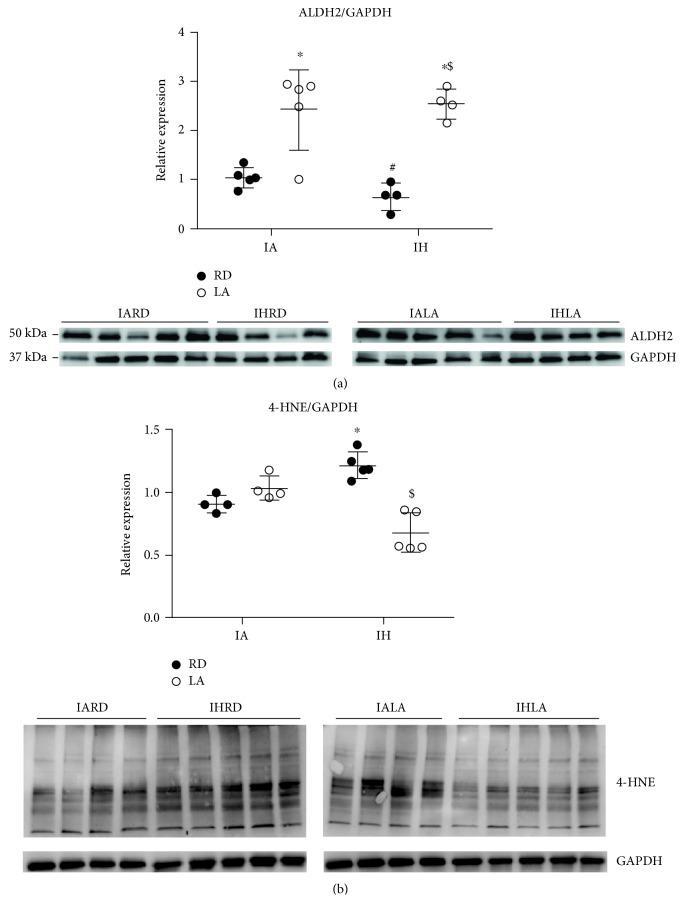
Aortic protein expression of ALDH2 (a) and 4-HNE protein adduct levels (b). Values are reported as mean ± SD and represent *n* = 5-6 mice. Statistical analysis was by two-way repeated measures ANOVA followed by Bonferroni post-hoc test. ^∗^*P* < 0.05 versus IARD, ^#^*P* < 0.05 versus IALA, and ^$^*P* < 0.05 versus IHRD. 4-HNE: 4-hydroxynonenal; ALDH2: aldehyde dehydrogenase 2; GAPDH: glyceraldehyde 3-phosphate dehydrogenase.

ALDH2 is a mitochondrial enzyme that metabolizes acetaldehyde and detoxifies reactive aldehydes, such as 4-HNE, that are generated from lipid peroxidation caused by oxidative stress [19]. Aortic expression of ALDH2 was not affected in mice exposed to CIH when compared to control (Figure 6(a)). However, expression of 4-HNE-modified proteins was higher in the CIH group. Treatment with ALA increased ALDH2 expression and decreased 4-HNE-modified protein expression in mice exposed to CIH (Figure 6(b)).

### 3.8. ALA Upregulated Antioxidant Enzymes and Blunted Inflammatory Cytokine Gene Expression in Mice Subjected to CIH

Aortic mRNA expression of inflammatory cytokines was increased in mice exposed to CIH when compared to control, while the expression of antioxidant genes was not affected (Table 2). Mice exposed to CIH and treated with ALA had increased gene expression of antioxidant enzymes (11- to 66-fold) and reduced expression of inflammatory cytokines (8- to 34-fold) when compared to mice exposed to CIH alone (Table 3).

## 4. Discussion

**Figure 7:**
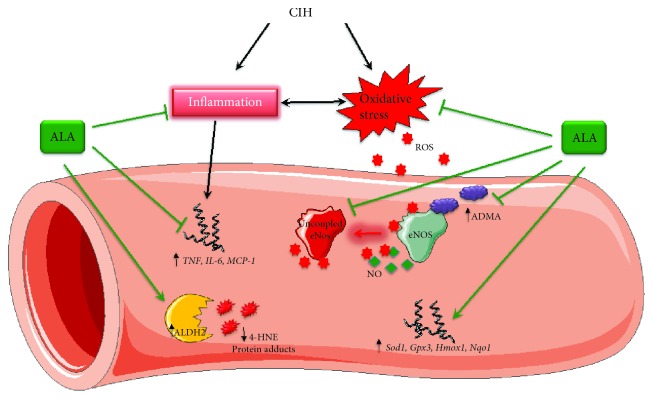
Summary of antioxidant and anti-inflammatory effects of ALA in mice exposed to CIH. 4-HNE: 4-hydroxynonenal; ADMA: asymmetric dimethylarginine; ALDH2: aldehyde dehydrogenase-2; ALA: alpha lipoic acid; CIH: chronic intermittent hypoxia; eNOS: endothelial nitric oxide synthase; Gpx3: glutathione peroxidase-3; Hmox1: heme oxygenase-1; IL-6: interleukin-6; MCP-1: monocyte chemoattractant protein-1; NO: nitric oxide; Nqo1: NAD(P)H dehydrogenase-1; ROS: reactive oxygen species; Sod1: superoxide dismutase-1; TNF: tumor necrosis factor alpha.

We demonstrated that dietary ALA treatment attenuated endothelial dysfunction in mice exposed to CIH as indicated by improvements in acetylcholine-induced vasodilation and basal nitric oxide production. We also show that (1) ALA decreased oxidative DNA damage and inflammatory marker levels in urine and plasma (2) prevented mRNA expression of inflammatory markers in aortic tissue, (3) increased mRNA expression of antioxidant enzymes in aortic tissue, and (4) maintained eNOS in a coupled state. Findings of this study are summarized in Figure 7.

OSA is associated with oxidative stress, inflammation, and endothelial dysfunction [3]. We and others reported endothelial dysfunction caused by CIH in mouse aorta [13] and other vascular beds such as rat cerebral and skeletal muscle arteries [20]. The extent of endothelial dysfunction depends on the intensity and duration of CIH [21]. Although CPAP can reverse endothelial dysfunction in OSA patients [22], patient adherence limits its use [23]. Treatment of OSA patients with intravenous injection of 0.5 g vitamin C acutely improved endothelial function (measured by flow-mediated dilation), suggesting a role for antioxidant treatment of OSA-related CVD [24]. This study, however, had a small sample size (*n* = 10), and treatment was only one injection of vitamin C without controlling for body weight. Treating rodents with xanthine oxidase [25] reduced endothelial dysfunction caused by CIH but had no effects on oxidative stress markers. We used ALA, a readily available, relatively safe agent, to improve endothelial function; this compound has both antioxidant and anti-inflammatory effects [26]. Our data show that ALA ameliorates endothelial dysfunction in mice subjected to CIH. Clinical and animal studies confirm that ALA improves endothelial function in several other diseases [8, 10, 27]. Conversely, ALA treatment in mice subjected to intermittent air resulted in reduced endothelial function when compared to intermittent air with regular diet. Some studies reported prooxidant effects of ALA only in animal studies, likely due to higher plasma concentrations than those after oral or intravenous infusion of ALA in humans [28, 29]. In our study, we did not observe any increase in oxidative stress or inflammation in ALA-treated control. We suggest that the inhibition of endothelium-dependent vasodilation is independent on ALA's prooxidant effect. The controversial outcomes of ALA treatment arise from the dose, route of administration, enantiomer used, disease, and duration of treatment [30]. It is essential to evaluate all these factors in clinical use to avoid the adverse effects of ALA.

Oxidative stress and inflammation are important mechanisms of endothelial dysfunction in OSA and CVD [31]. Oxidative stress is characterized by an imbalance between the antioxidant system and prooxidant systems leading to accumulation of reactive oxygen species (ROS). CIH in OSA leads to increased ROS production and impairment of antioxidant capacity [32]. Increased ROS production interacts with nitric oxide to decrease its bioavailability and produces a potent reactive nitrogen-free radical (peroxynitrite) that oxidizes lipids, proteins, and DNA [33]. A recent study of OSA patients concluded that 2 months of CPAP treatment did not reduce oxidative stress markers despite CPAP adherence [34]. On the other hand, NO concentrations measured during sleep in 8 OSA patients were significantly lower when compared to 6 snorers and 6 normal adults and treatment with CPAP restored NO levels and increased levels of L-arginine, the substrate for eNOS [35]. Our study demonstrated that urinary levels of 8-OHdG are higher in mice subjected to CIH, similar to findings in OSA patients [36]. Treatment with dietary ALA not only lowered systemic oxidative stress but also increased aortic mRNA levels of antioxidant enzymes in mice exposed to CIH. Indeed, ALA can directly act as an antioxidant, regenerating and maintaining endogenous antioxidants and activating nuclear factor- (erythroid-derived 2-) like 2 (Nrf2) (a key transcription factor that mediates the expression of antioxidant and detoxification genes regulated by the antioxidant response element (ARE)) [37].

ADMA is a naturally occurring L-arginine analog derived from proteolysis of methylated protein, and the enzyme DDAH prevents its accumulation. However, in pathological conditions such as chronic kidney failure [38], ADMA levels are elevated and can compete with L-arginine for the binding site in the active center of NOS and thus inhibit the production of nitric oxide [18]. ADMA is an independent risk factor for coronary heart disease according to the multicenter Coronary Artery Risk Determination investigating the Influence of ADMA Concentration (CARDIAC) study [39]. Others have reported that ADMA levels are elevated in patients with OSA and mice exposed to CIH [13, 40]. In our study, treatment of mice exposed to CIH with ALA decreased levels of ADMA without affecting DDAH-2 expression in the aorta. Oxidative stress can reduce the activity and can cause ADMA levels to increase [41]. ALA as an antioxidant might have protected DDAH-2 from oxidation, which could account for the decreased levels of ADMA in the CIH group.

ALDH2 is the mitochondrial form of aldehyde dehydrogenase responsible for metabolism of toxic aldehydes and ROS-generated aldehyde adducts that can adduct with lipids, proteins, and DNA, leading to their inactivation [19, 42]. ALDH2 activity involves cysteine thiol groups that are susceptible to oxidative stress and so render the enzyme inactive [43]. ALA (and its reduced form) can restore the activity of oxidized ALDH2 [44, 45]. Our results show that ALDH2 expression was not affected by CIH but treatment of mice exposed to CIH with ALA significantly increased ALDH2 expression in aortic tissue. Furthermore, CIH increased 4-HNE-modified protein expression in aorta but treatment with ALA decreased the expression these proteins significantly, indicating that ALA may have enhanced 4-HNE detoxification through increasing ALDH2 activity.

Inflammation is prominent in OSA and is responsible for initiation of atherosclerosis in CVD [46]. Oxidative stress activates transcription factor NF-*κ*B, causing it to translocate to the nucleus where it initiates the transcription of various inflammatory cytokines such as interlukin-6 (IL-6) and TNF-*α* and endothelial adhesion molecules [47]. Recent clinical trials reported that CPAP did not improve levels of C-reactive protein (CRP), IL-6, and TNF-*α* in OSA patients [48, 49]. We show that plasma levels of TNF-*α* and mRNA expression of inflammatory cytokines such as TNF-*α*, IL-6, and monocyte attractant protein 1 (MCP-1) are increased by CIH and that dietary ALA reversed systemic and aortic inflammation in mice subjected to CIH. This effect is likely due to the ability of ALA to prevent the translocation of NF-*κ*B [50] independent of its antioxidant mechanisms [51].

OSA uncouples eNOS in the vasculature, causing production of superoxide anion instead of nitric oxide. Oxidative stress leads to the oxidation of tetrahydrobiopterin (BH_4_), an essential cofactor essential for NO production, leading to eNOS uncoupling. Supplementation with BH_4_ reverses endothelial dysfunction in OSA patients [52]. We evaluated eNOS uncoupling in aortic sections by measuring the fluorescence of the superoxide-sensitive dye (DHE) before and after incubation with L-NAME (eNOS inhibitor). Increased fluorescence in control mice after L-NAME indicates decreased nitric oxide availability for interaction with superoxide anion. On the other hand, decreased fluorescence after L-NAME incubation indicates blockage of uncoupled eNOS due to lower amounts of superoxide anion, as seen in mice subjected to CIH. However, ALA treatment in mice subjected to CIH increased fluorescence after L-NAME incubation, indicating that eNOS was preserved its coupled state.

There are several limitations to our study. Firstly, the CIH model used does not incorporate airway obstruction and thus lacks the intrathoracic pressure swings and hypercapnia that occurs in humans with OSA. Secondly, the CIH profile used (5-6%) is very severe and potentially induced the vascular dysfunction in our study whereas other studies have shown that less severe CIH (10%) may be vasculoprotective [53, 54]. Thirdly, we only used male mice in our study and studies on the effects of CIH in female mice are warranted.

## 5. Conclusion

In summary, chronic IH increases systemic and vascular oxidative stress and inflammation, resulting in reduced nitric oxide bioavailability and endothelial dysfunction. Treating mice with ALA had multiple effects that preserved nitric oxide-dependent dilation and reduced inflammation and oxidative stress, suggesting that it may be a potentially promising therapy to improve cardiovascular health in patients with obstructive sleep apnea.

## Figures and Tables

**Table 1:** Characteristics of mice exposed to chronic intermittent hypoxia and treated with alpha lipoic acid compared to controls.

Group	Body weight (g)	Epidydimal fat (g)	Fasting blood glucose (mmol/L)	Plasma insulin (pg/mL)
IARD	36.7 ± 1.7	1.8 ± 0.3	7.9 ± 0.5	0.35 ± 0.05
IALA	33.3 ± 3.1	1.4 ± 0.6	7.5 ± 1.2	0.36 ± 0.04
IHRD	36.5 ± 4.1	1.9 ± 0.6	6.7 ± 0.9	0.44 ± 0.06
IHLA	33.7 ± 1.5	1.4 ± 0.2	6.4 ± 0.2	0.32 ± 0.07^$^

Values are displayed as mean ± SD and represent *n* = 5 − 6 mice. Statistical analysis was done by two-way repeated measures ANOVA followed by Bonferroni post-hoc test. ^∗^*P* < 0.05 versus IARD, ^#^*P* < 0.05 versus IALA, and ^$^*P* < 0.05 versus IHRD. IARD: intermittent air with regular diet; IALA: intermittent air with alpha lipoic acid; IHRD: intermittent hypoxia with regular diet; IHLA: intermittent hypoxia with alpha lipoic acid.

**Table 2:** Fold changes of gene expression in aortic tissue of mice exposed to intermittent hypoxia and treated with ALA compared to control.

	Gene symbol	Name of gene	IHRD	IHLA	IALA
Inflammatory cytokines	*IL-6*	Interleukin 6	4	46^∗^	9
	*TNF*	Tumor necrosis factor alpha	1	34^∗^	10
	*MCP-1*	Monocyte chemoattractant protein 1	2	1	1
	*Ifng*	Interferon gamma	36^∗^	5	2
Adhesion molecules	*Icam1*	Intracellular adhesion molecule 1	64	2	1
	*Vcam1*	Vascular cell adhesion molecule 1	56	36^∗^	16^∗^
Antioxidant enzymes	*Sod1*	Superoxide dismutase 1	2	74^∗^	9
	*Gpx3*	Glutathione peroxidase 3	1	89^∗^	4
	*Hmox1*	Heme oxygenase 1	−2^∗^	−1	−1
	*Nqo1*	NAD(P)H dehydrogenase	3^∗^	3	2
ROS-producing enzymes	*Nox1*	NADPH oxidase 1	43	5	1
	*Nox4*	NADPH oxidase 4	8	4	2

Student's unpaired *t*-test was performed on ^ΔΔ^*C*_*T*_ values between groups; values are expressed as mean ± SD (*n* = 5 − 6 mice) (^∗^*P* < 0.05 versus IARD). Fold regulation represents fold change in a biologically meaningful way.

**Table 3:** Fold changes of gene expression in aortic tissue of mice exposed to chronic intermittent hypoxia compared to mice treated with alpha lipoic acid.

	Gene symbol	Name of gene	Fold regulation in IHRD vs. IHLA
Inflammatory cytokines	*IL-6*	Interleukin 6	−7^∗^
	*TNF*	Tumor necrosis factor alpha	−8^∗^
	*MCP-1*	Monocyte chemoattractant protein 1	−34^∗^
	*Ifng*	Interferon gamma	1
Adhesion molecules	*Icam1*	Intracellular adhesion molecule 1	−1
	*Vcam1*	Vascular cell adhesion molecule 1	2
Antioxidant enzymes	*Sod1*	Superoxide dismutase 1	11^∗^
	*Gpx3*	Glutathione peroxidase 3	29^∗^
	*Hmox1*	Heme oxygenase 1	38^∗^
	*Nqo1*	NAD(P)H dehydrogenase	66^∗^
ROS-producing enzymes	*Nox1*	NADPH oxidase 1	−2
	*Nox4*	NADPH oxidase 4	−2

Student's unpaired *t-*test was performed on ^ΔΔ^*C*_*T*_ values between groups; values are expressed as mean ± SD (*n* = 5-6 mice), ^∗^*P* < 0.05 versus IHRD. Fold regulation represents fold change in a biologically meaningful way.

## Data Availability

The data used to support the findings of this study are available from the corresponding author upon request.

## References

[1] Badran M., Ayas N., Laher I. (2014). Insights into obstructive sleep apnea research. *Sleep Medicine*.

[2] Young T., Finn L., Peppard P. E. (2008). Sleep disordered breathing and mortality: eighteen-year follow-up of the Wisconsin sleep cohort. *Sleep*.

[3] Golbidi S., Badran M., Ayas N., Laher I. (2012). Cardiovascular consequences of sleep apnea. *Lung*.

[4] Shafazand S., Patel S. R. (2014). Effect of CPAP on blood pressure in patients with obstructive sleep apnea and resistant hypertension. *Journal of Clinical Sleep Medicine*.

[5] Weaver T. E., Grunstein R. R. (2008). Adherence to continuous positive airway pressure therapy: the challenge to effective treatment. *Proceedings of the American Thoracic Society*.

[6] McEvoy R. D., Antic N. A., Heeley E. (2016). CPAP for prevention of cardiovascular events in obstructive sleep apnea. *The New England Journal of Medicine*.

[7] Golbidi S., Badran M., Laher I. (2011). Diabetes and alpha lipoic acid. *Frontiers in Pharmacology*.

[8] Heinisch B. B., Francesconi M., Mittermayer F. (2010). Alpha-lipoic acid improves vascular endothelial function in patients with type 2 diabetes: a placebo-controlled randomized trial. *European Journal of Clinical Investigation*.

[9] Hager K., Kenklies M., McAfoose J., Engel J., Münch G., Gerlach M., Deckert J., Double K., Koutsilieri E. (2007). *α*-Lipoic acid as a new treatment option for Alzheimer’s disease — a 48 months follow-up analysis. *Neuropsychiatric Disorders An Integrative Approach*.

[10] Lee W. R., Kim A., Kim K. S. (2012). Alpha-lipoic acid attenuates atherosclerotic lesions and inhibits proliferation of vascular smooth muscle cells through targeting of the Ras/MEK/ERK signaling pathway. *Molecular Biology Reports*.

[11] Yi X., Maeda N. (2006). *α*-Lipoic acid prevents the increase in atherosclerosis induced by diabetes in apolipoprotein E–deficient mice fed high-fat/low-cholesterol diet. *Diabetes*.

[12] Suh J. H., Moreau R., Heath S. H., Hagen T. M. (2005). Dietary supplementation with (*R*)-*α*-lipoic acid reverses the age-related accumulation of iron and depletion of antioxidants in the rat cerebral cortex. *Redox Report*.

[13] Badran M., Abuyassin B., Golbidi S., Ayas N., Laher I. (2016). Uncoupling of vascular nitric oxide synthase caused by intermittent hypoxia. *Oxidative Medicine and Cellular Longevity*.

[14] Sallam N., Fisher A., Golbidi S., Laher I. (2011). Weight and inflammation are the major determinants of vascular dysfunction in the aortae of db/db mice. *Naunyn-Schmiedeberg’s Archives of Pharmacology*.

[15] Badran M., Golbidi S., Devlin A., Ayas N., Laher I. (2014). Chronic intermittent hypoxia causes endothelial dysfunction in a mouse model of diet-induced obesity. *Sleep Medicine*.

[16] Lee W. J., Song K. H., Koh E. H. (2005). Alpha-lipoic acid increases insulin sensitivity by activating AMPK in skeletal muscle. *Biochemical and Biophysical Research Communications*.

[17] Cakatay U. (2006). Pro-oxidant actions of alpha-lipoic acid and dihydrolipoic acid. *Medical Hypotheses*.

[18] Badran M., Golbidi S., Ayas N., Laher I. (2015). Nitric oxide bioavailability in obstructive sleep apnea: interplay of asymmetric dimethylarginine and free radicals. *Sleep Disorders*.

[19] Guo J. M., Liu A. J., Zang P. (2013). ALDH2 protects against stroke by clearing 4-HNE. *Cell Research*.

[20] Phillips S. A., Olson E. B., Morgan B. J., Lombard J. H. (2004). Chronic intermittent hypoxia impairs endothelium-dependent dilation in rat cerebral and skeletal muscle resistance arteries. *American Journal of Physiology. Heart and Circulatory Physiology*.

[21] Savransky V., Nanayakkara A., Li J. (2007). Chronic intermittent hypoxia induces atherosclerosis. *American Journal of Respiratory and Critical Care Medicine*.

[22] Schwarz E. I., Puhan M. A., Schlatzer C., Stradling J. R., Kohler M. (2015). Effect of CPAP therapy on endothelial function in obstructive sleep apnoea: a systematic review and meta-analysis. *Respirology*.

[23] Ghosh D., Allgar V., Elliott M. W. (2013). Identifying poor compliance with CPAP in obstructive sleep apnoea: a simple prediction equation using data after a two week trial. *Respiratory Medicine*.

[24] Grebe M., Eisele H. J., Weissmann N. (2006). Antioxidant vitamin C improves endothelial function in obstructive sleep apnea. *American Journal of Respiratory and Critical Care Medicine*.

[25] Dopp J. M., Philippi N. R., Marcus N. J. (2011). Xanthine oxidase inhibition attenuates endothelial dysfunction caused by chronic intermittent hypoxia in rats. *Respiration*.

[26] Shay K. P., Moreau R. F., Smith E. J., Smith A. R., Hagen T. M. (1790). Alpha-lipoic acid as a dietary supplement: molecular mechanisms and therapeutic potential. *Biochimica et Biophysica Acta (BBA) - General Subjects*.

[27] Xiang G. D., Pu J. H., Sun H. L., Zhao L. S. (2010). Alpha-lipoic acid improves endothelial dysfunction in patients with subclinical hypothyroidism. *Experimental and Clinical Endocrinology & Diabetes*.

[28] Cakatay U., Kayali R., Sivas A., Tekeli F. (2005). Prooxidant activities of alpha-lipoic acid on oxidative protein damage in the aging rat heart muscle. *Archives of Gerontology and Geriatrics*.

[29] Rochette L., Ghibu S., Richard C., Zeller M., Cottin Y., Vergely C. (2013). Direct and indirect antioxidant properties of *α*-lipoic acid and therapeutic potential. *Molecular Nutrition & Food Research*.

[30] Gomes M. B., Negrato C. A. (2014). Alpha-lipoic acid as a pleiotropic compound with potential therapeutic use in diabetes and other chronic diseases. *Diabetology and Metabolic Syndrome*.

[31] Lavie L. (2015). Oxidative stress in obstructive sleep apnea and intermittent hypoxia--revisited--the bad ugly and good: implications to the heart and brain. *Sleep Medicine Reviews*.

[32] Lavie L., Lavie P. (2009). Molecular mechanisms of cardiovascular disease in OSAHS: the oxidative stress link. *The European Respiratory Journal*.

[33] Badran M., Ayas N., Laher I. (2014). Cardiovascular complications of sleep apnea: role of oxidative stress. *Oxidative Medicine and Cellular Longevity*.

[34] Paz Y., Mar H. L., Hazen S. L. (2016). Effect of continuous positive airway pressure on cardiovascular biomarkers: the sleep apnea stress randomized controlled trial. *Chest*.

[35] Lavie L., Hefetz A., Luboshitzky R., Lavie P. (2003). Plasma levels of nitric oxide and L-arginine in sleep apnea patients: effects of nCPAP treatment. *Journal of Molecular Neuroscience*.

[36] Yamauchi M., Nakano H., Maekawa J. (2005). Oxidative stress in obstructive sleep apnea. *Chest*.

[37] Rochette L., Ghibu S., Muresan A., Vergely C. (2015). Alpha-lipoic acid: molecular mechanisms and therapeutic potential in diabetes. *Canadian Journal of Physiology and Pharmacology*.

[38] Vallance P., Leone A., Calver A., Collier J., Moncada S. (1992). Accumulation of an endogenous inhibitor of nitric oxide synthesis in chronic renal failure. *The Lancet*.

[39] Schulze F., Lenzen H., Hanefeld C. (2006). Asymmetric dimethylarginine is an independent risk factor for coronary heart disease: results from the multicenter Coronary Artery Risk Determination investigating the Influence of ADMA Concentration (CARDIAC) study. *American Heart Journal*.

[40] Barceló A., de la Peña M., Ayllón O. (2009). Increased plasma levels of asymmetric dimethylarginine and soluble CD40 ligand in patients with sleep apnea. *Respiration*.

[41] Ito A., Tsao P. S., Adimoolam S., Kimoto M., Ogawa T., Cooke J. P. (1999). Novel mechanism for endothelial dysfunction: dysregulation of dimethylarginine dimethylaminohydrolase. *Circulation*.

[42] Hill B. G., Bhatnagar A. (2009). Beyond reactive oxygen species: aldehydes as arbitrators of alarm and adaptation. *Circulation Research*.

[43] Daiber A., Oelze M., Coldewey M. (2004). Oxidative stress and mitochondrial aldehyde dehydrogenase activity: a comparison of pentaerythritol tetranitrate with other organic nitrates. *Molecular Pharmacology*.

[44] He L., Liu B., Dai Z. (2012). Alpha lipoic acid protects heart against myocardial ischemia-reperfusion injury through a mechanism involving aldehyde dehydrogenase 2 activation. *European Journal of Pharmacology*.

[45] Wenzel P., Hink U., Oelze M. (2007). Role of reduced lipoic acid in the redox regulation of mitochondrial aldehyde dehydrogenase (ALDH-2) activity. Implications for mitochondrial oxidative stress and nitrate tolerance. *Journal of Biological Chemistry*.

[46] Kritikou I., Basta M., Vgontzas A. N. (2014). Sleep apnoea, sleepiness, inflammation and insulin resistance in middle-aged males and females. *European Respiratory Journal*.

[47] Van der Heiden K., Cuhlmann S., Luong L. A., Zakkar M., Evans P. C. (2010). Role of nuclear factor *κ*B in cardiovascular health and disease. *Clinical Science*.

[48] Kohler M., Ayers L., Pepperell J. C. (2009). Effects of continuous positive airway pressure on systemic inflammation in patients with moderate to severe obstructive sleep apnoea: a randomised controlled trial. *Thorax*.

[49] Stradling J. R., Craig S. E., Kohler M. (2015). Markers of inflammation: data from the MOSAIC randomised trial of CPAP for minimally symptomatic OSA. *Thorax*.

[50] Li G., Fu J., Zhao Y., Ji K., Luan T., Zang B. (2015). Alpha-lipoic acid exerts anti-inflammatory effects on lipopolysaccharide-stimulated rat mesangial cells via inhibition of nuclear factor kappa B (NF-*κ*B) signaling pathway. *Inflammation*.

[51] Ying Z., Kampfrath T., Sun Q., Parthasarathy S., Rajagopalan S. (2011). Evidence that *α*-lipoic acid inhibits NF-*κ*B activation independent of Its antioxidant function. *Inflammation Research*.

[52] Varadharaj S., Porter K., Pleister A. (2015). Endothelial nitric oxide synthase uncoupling: a novel pathway in OSA induced vascular endothelial dysfunction. *Respiratory Physiology & Neurobiology*.

[53] Jackman K. A., Zhou P., Faraco G. (2014). Dichotomous effects of chronic intermittent hypoxia on focal cerebral ischemic injury. *Stroke*.

[54] Manukhina E. B., Jasti D., Vanin A. F., Downey H. F. (2011). Intermittent hypoxia conditioning prevents endothelial dysfunction and improves nitric oxide storage in spontaneously hypertensive rats. *Experimental Biology and Medicine*.

